# Pseudocereal-Based Functional Beverages: Main Properties and Nutritional Evaluation with an Emphasis on Amino Acid Content: A Review

**DOI:** 10.3390/foods14122080

**Published:** 2025-06-12

**Authors:** Dorottya Krisztina Vajdovich, Éva Csajbókné Csobod, Csilla Benedek

**Affiliations:** 1 Health Sciences Division, Doctoral College, Semmelweis University, 1085 Budapest, Hungary; vajdovich.dorottya@stud.semmelweis.hu; 2 Department of Dietetics and Nutritional Sciences, Faculty of Health Sciences, Semmelweis University, 1085 Budapest, Hungary; csajbokne.csobod.eva@semmelweis.hu

**Keywords:** functional beverages, pseudocereals, protein digestibility, amino acids

## Abstract

The demand for functional beverages has increased significantly in recent years as society places more and more emphasis on healthy lifestyles and disease prevention. Functional beverages may contain various health-promoting, bioactive compounds (e.g., antioxidants, vitamins, minerals, prebiotics, probiotics, proteins, etc.). These ingredients originate from sources including fruits (e.g., red berries), vegetables (e.g., spinach), nuts (e.g., flaxseeds), and herbs (e.g., turmeric), or can be added as separate components (e.g., prebiotics). Their nutritional properties qualify pseudocereals (quinoa, buckwheat, and amaranth) as ideal bases for functional beverages. They are high in antioxidants (e.g., polyphenols), vitamins (e.g., folate), and minerals (e.g., iron). Their high protein content (5.7–25.3%, about three times higher than that of maize) improves the nutritional profile of plant-based drinks. They have a balanced protein and amino acid composition, as they contain all the essential amino acids (among which lysine is present in high amounts) and are gluten-free. The in vitro protein digestibility of pseudocereals is also outstanding (PDCAAS: quinoa (0.85), amaranth (0.70), and buckwheat (0.78), while those for wheat, rice, and maize are 0.42, 0.56, and 0.47, respectively). Given these benefits, trends in producing and consuming plant-based, especially pseudocereal-based, functional beverages are highlighted in the present review.

## 1. Introduction

The increasing health-awareness of our society and the trends promoting lifelong well-being have led to a focus on improving nutrition [1], making people more open to foods that have a positive impact on the body, help weight control, reduce the incidence or slow the progression of chronic, lifestyle-related, non-communicable diseases, strengthen the immune system, etc. [2].

Consumers now recognize the strong correlation between the diet they follow and its positive health-related outcomes. In addition, hectic, irregular lifestyles, inadequate physical activity, high consumption of convenience foods with unfavorable ingredients, and health deterioration due to self-medication activities (including fad diets) have boosted the popularity of functional foods [3]. Studies have shown that these foods support health, prevent diseases, and have emerged as catalysts for good nutrition [1,4]. A key feature is that they can be marketed as food products only, i.e., they are not considered as medicines at any level [4,5]. The functional food sector includes primarily probiotics, prebiotics, antioxidants, plant supplements, dietary fibers, minerals, and other phytochemicals [2]. To exert their benefits, functional foods need to be consumed as part of the normal diet [6].

A large group of “functional foods” are “functional drinks” or “functional beverages”, whose market is expected to grow by 7.5% by the end of 2024, justifying the increased demand [3,7]. This is presumably due to innovations in dietetics and nutrition research in recent years. A functional beverage is defined as “any non-alcoholic beverage that provides additional health benefits through the incorporation of a bioactive component derived from plant, animal, marine or microorganisms” [3,8]. The concept of “functional beverages” or “functional drinks” differs from that of functional foods because it is basically a liquid that hydrates the body while also having other specific health benefits as well [3].

There are several types of functional beverages, including fruit and vegetable beverages, milk-based or dairy drinks, pre- and probiotic drinks, sports drinks, energy drinks, phytoactive-fortified drinks, beauty drinks, cognitive- and immune-enhancing drinks, etc. [3,9]. Functional beverage ingredients can include ingredients with biologically active properties, such as vegetables, fruits, herbs, coffee, cocoa, tea, sweeteners, dairy products, pulses, cereals, or even pseudocereals. In most cases, additional bioactive ingredients are also added (e.g., prebiotics, probiotics, dietary fibers, vitamins, minerals, etc.) [3].

As the world population is estimated to increase by 1.5 billion by 2050, it is important to find new sources of healthy nutrients to meet the need of protein sources. Also, there is a clear trend towards a more health-conscious and sustainable protein intake which has produced a shift towards plant-based protein sources [10]. Healthy protein intake, therefore, highly depends on the protein quality of protein-containing foods. However, in order to understand the quality of the protein content, it is important to consider the amino acid composition and the digestibility of proteins, as well as the absorption of amino acids and peptides during protein digestion. A key determinant of an adequate protein utilization is the adequacy of the protein to meet human metabolic needs. The digestibility of proteins is influenced by several factors, e.g., the presence of antinutritional compounds, absorption process, digestion conditions, etc. There are several methods that measure digestibility; these include the protein efficiency ratio (PER), the protein digestibility corrected amino acid score (PDCAAS), and the digestible indispensable amino acid score (DIAAS) [11].

Although plant-based diets have many health benefits, they do not always reach the nutritional quality of animal products (e.g., dairy, eggs, meat, fish, etc.) in terms of protein quality [11]. Thus, the consumption of pseudocereals gains importance due to their protein content, combined with their positive health effects and biologically active compounds [12]. In general, compared to cereals, pseudocereals are lower in carbohydrates, but higher in protein, fat, and fiber [10]. Since pseudocereals are not botanically related to cereals, they have a different amino acid composition [11]. They have a well-balanced amino acid profile, being high in methionine, cysteine, and lysine. In addition, they are rich in bioactive compounds, such as unsaturated fatty acids, dietary fibers, flavonoids, lignans, antioxidant compounds, polyphenols, phytosterols, vitamins, and minerals [11].

The aim of this study is to provide an overview of the importance and the main ingredient groups of functional beverages. It highlights the potential of pseudocereals as plant-based protein sources in functional beverages, focusing on their high digestible protein content and balanced amino acid composition.

For the literature search, PubMed, Science Direct, Web of Science, Google Scholar (partly), Semantic Scholar, and relevant journal databases (e.g., *Foods*, *Nutrients*, *Functional Foods*, and *Food Chemistry*) were used. Keywords, like “functional foods”, “functional beverages”, “functional drinks”, “functional beverages AND protein”, “plant-based beverages AND protein”, “pseudo-cereals”, “pseudocereals”, and “pseudocereal-based functional beverages”, etc., were applied for the search.

## 2. The Definition of Functional Foods

Over the past decade, particular attention has been paid to foods that possess functional properties in addition to their nutritional content [12]. This is particularly noticeable in the aftermath of the COVID-19 pandemic, as society shares the common goal of combating epidemics by maintaining general good health [2].

Today, the food industry is committed to producing foods with additional nutritional and health benefits [2]. The health benefits of functional foods and beverages have also been highlighted by the World Health Organization (WHO) [3].

Functional foods are also called “foods of the future” [2], because they are defined as foods that, in addition to their existing essential nutritional value, contain additional beneficial components that may provide health benefits to the body yet are still considered foods [1,4,13]. In a more precise formulation, foods belonging to the functional category are foods with the potential to promote health and to fulfil physiological functions of the individual, but not to cure diseases [4].

The Functional Food Centre (Dallas, USA) has revised the definition of functional foods [5], defining them as natural or industrially processed foods that contain biologically active compounds with proven beneficial effects and that, when administered in appropriate, non-toxic doses, may help prevent, treat, or control chronic diseases or their symptoms, as demonstrated along defined biomarkers [4].

However, the precise definition of functional foods is still incomplete, the problem being that it is a very broad concept and effectively excludes only a small group of foods, i.e., those foods that are present in large quantities in the “Western-style” diet (e.g., foods containing added sugars, foods containing saturated or trans fats, and alcoholic beverages). A more specific definition is, therefore, needed, which could refer to functional foods as being newly developed foods that have been specifically formulated to contain health-promoting or disease-preventing substances or live microorganisms. The concentration of active substances present in these foods should reach a level that is both safe and sufficient to induce the physiological benefit. The added components may include nutrients, dietary fibers, phytochemicals, other bioactive substances, and probiotics. The Institute of Food Technologists has defined functional foods in a similar way, while noting that these foods are “novel”, which means that, for example, yoghurt or refined cereals (which contain vitamins B) do not fall into the category of functional foods. However, this definition needs further discussion [14].

Functional foods are clearly different from medicines, pharmaceuticals, and food supplements. These foods have health-promoting effects that target disease prevention, but they may also be used for therapeutic purposes [6].

## 3. Criteria of Functional Foods

Functional foods come under various names, such as “designer foods” or “nutraceuticals”, and the criteria for these foods are still very diverse, taking into account the historical background, culture, and regulations of the country concerned [4]. In order to reach the consumer society, functional foods must comply with a number of regulations and food safety standards [2].

However, summarizing the many definitions, a functional food must meet four main criteria, as follows [4,5]:

1. The food has certain health benefits;
2. The nature of the food (e.g., does it contain conventional or modified, fortified, or naturally occurring ingredients? Is it overdosed?);
3. Nutritional effectiveness (e.g., does it go beyond conventional foods?);
4. Consumption pattern (e.g., does it take cultural habits into account? Can it be integrated into the daily eating pattern?).

Functional food ingredients are mostly a variety of biologically active compounds, such as phytochemicals, essential fatty acids, vitamins, and minerals. Thanks to these biologically active ingredients, occurring naturally or added in pure form, functional foods can be distinguished from conventional foods, as they go beyond basic nutritional factors and contribute effectively to health promotion [1]. Functional foods are, therefore, designed to promote health, increase overall well-being and reduce the risk of diseases [13].

Scientific evidence on functional foods has been presented in a number of studies and clinical trials that support the links between functional food consumption and improvements in certain health parameters. Research on functional foods has examined properties that demonstrate the effects of these specific foods on cardiovascular function, immune response, cognitive performance, and metabolic balance [1].

## 4. Categories of Functional Foods

Studies have classified functional foods into several categories in order to increase the amount of research on this topic, to facilitate personalized diet planning, or to educate consumers about the benefits of these foods [1].

For the classification of functional foods, the following six categories have been identified by the Functional Food Centre (Dallas, TX, USA) [4,5]:

1. Natural foods—these occur naturally, without any modification or processing;
2. Fortified foods—functional ingredients are added;
3. Removal of a harmful or abnormal component—which may have a negative effect on human health;
4. Modification of technological processes—enhancing the formation of bioactive components during the process (e.g., fermentation);
5. Special growing conditions, genetic manipulation or new varieties—special feeding, genetically modified products;
6. Combinations of the above.

Functional foods can include certain vegetables, fruits, cereals, pseudocereals, nuts, oilseeds, and pulses, but also dairy products, certain beverages, fish, herbs, and spices, each of which has unique properties and can be targeted to improve health [1].

## 5. Functional Beverages

A large group among functional foods is the group of functional beverages. Functional beverages may contain added vitamins, minerals, herbs, or other bioactive compounds. The specific purpose of these functional “additives” is to improve health or a particular, specific function of the body. Such drinks include, for example, sports drinks, energy drinks, and vitamin-enriched waters. Certain functional herbal teas, juices, and smoothies serving general well-being are also included here [3].

Functional beverages are popular because they are easy to be fortified with bioactive components, have lower production costs, are easier to preserve and store, and are more acceptable to consumers [15]. They have a role to play in the improvement of immune, gastrointestinal, and cardiovascular conditions through their functional ingredients [16,17].

Functional beverages are dairy-, fruit and vegetable-, pulse-, coffee-, tea-, or cereal-based drinks with functional purposes, such as boosting energy, slowing down the aging process, reducing fatigue or stress, or specifically preventing or even treating lifestyle-related diseases [18]. The effects of functional drinks were observed in vitro and in vivo. Under in vitro conditions, cellular glucose uptake was improved and the enzymes α-glucosidase and α-amylase were inhibited. In nude mice studied in vivo, it was observed that functional beverage consumption improved body weight, reduced insulin resistance, improved ß-cell function, reduced blood glucose, reduced oxidative stress, improved lipid profile and reduced blood pressure [15].

An important part of their appeal is the quality of their packaging, which is designed specifically to meet consumer needs, taking into account the ease of transport and storage, as well as cost-effective production. All these factors have led to the spreading of functional drinks on the market [18].

### 5.1. Types of Functional Drinks

**Figure 1:**
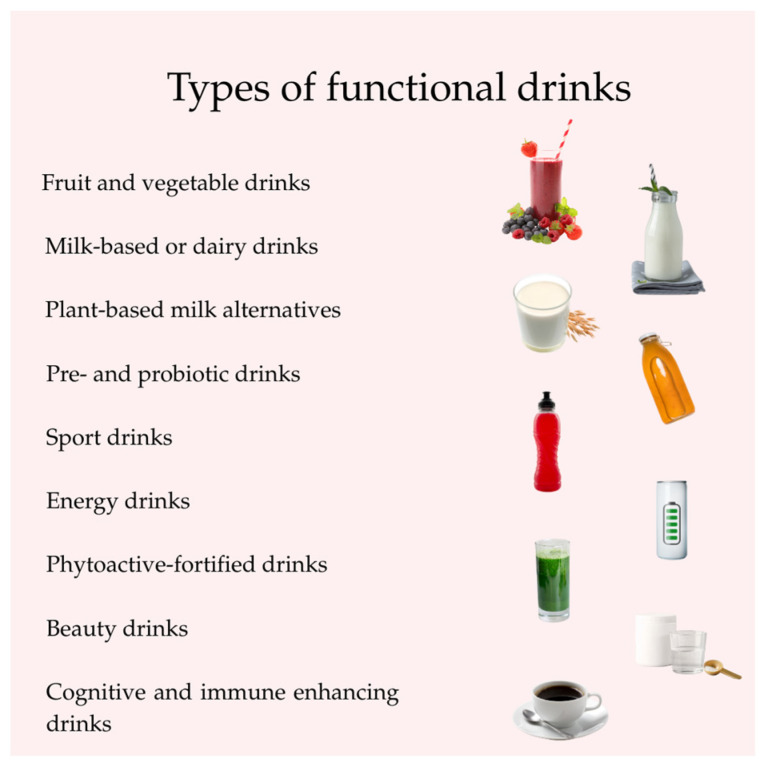
Types of functional drinks.

As mentioned earlier, there are several types of functional drinks. Some types are explained separately here and are shown in Figure 1 [3,9]:

#### 5.1.1. Fruit and Vegetable Drinks

Fruits and vegetables are key components of a healthy diet because, in addition to being lower in energy density, they are high in fiber and contain many bioactive components (e.g., antioxidants, vitamins, minerals, and dietary fiber). They are an excellent source of vitamin C [9]. They also help prevent and treat chronic non-communicable diseases, such as heart disease, cancer, diabetes, and obesity, and help to manage micronutrient deficiencies [15].

#### 5.1.2. Milk-Based or Dairy Drinks

Milk-based functional drinks can include fresh milk, fermented milk, and yoghurt drinks as primary sources of probiotics [15]. When herbal extracts were added to yoghurts, their physicochemical properties, synthesis, water-holding capacity, and antioxidant properties were improved [9].

#### 5.1.3. Plant-Based Milk Alternatives

The demand for plant-based beverages is growing globally, with a 10.18% growth rate between 2020 and 2024, and this is likely due to the increasing number of people following a dairy-free diet. Due to other considerations, it is expected that the market for plant-based beverages will grow by 15% from 2023 to 2028 [19]. According to Plamada et al., there has been a significantly growing need for plant-based dairy substitutes over the last decade. Sales of plant-based milk substitutes increased by 9% in 2018, while sales of cow’s milk decreased by 6%. Some research shows that young people who value sustainable eating have healthier eating habits than those who do not [20].

The main reasons for this phenomenon are ethical concerns, concerns for environmental sustainability (reducing greenhouse gas emissions), and health-conscious lifestyles. In most cases, the production of plant-based beverages is much less energy-intensive, less polluting, and more sustainable than dairy production [21].

Plant-based milk alternatives are water-soluble extracts of the raw plant materials used, including cereals (rice, oats, millet, brown rice), pseudocereals (quinoa, amaranth, buckwheat), oilseeds (sesame, sunflower), nuts (hazelnuts, almonds, Brazil nuts, cashew nuts, walnuts), and pulses (chickpeas, peas, soya beans), which, once homogenized, will give a similar appearance to normal milk after grounding, milling, filtering, and then re-extracting [16]. As a result, the size of the particles is distributed between 5 and 20 μm [19]. At the same time, however, their organoleptic properties, shelf life, and heat stability are different from those of dairy milk [16,20].

Oats have excellent nutritional content, but the disadvantage of oats in plant-based milk alternatives is that they have a high starch content (about 60%), which results in a highly viscous gelatinous mass when the aqueous emulsion is heated. To avoid this, it is advisable to hydrolyze the starch beforehand [22].

Almonds, cashew nuts, and Brazil nuts are the most suitable nuts for use as plant-based milk replacers, while sesame and sunflower seeds are the most suitable oilseeds in this case [22].

Legumes are mostly found in the form of plant-based milk replacers or as an added source of protein in functional drinks. Soybeans, peas, and chickpeas are most commonly used. Legumes also have the advantage of being a source of protein, carbohydrates, minerals, vitamins, and fiber. Compared to other legumes, chickpeas contain few antinutrients, have low allergenicity, are easily digestible and are a source of iron. Chickpea protein is also superior compared to other legumes due to its easy digestibility and higher bioavailability. The amino acid composition of soybean is the most favorable of all legumes, but it also has a high allergenicity and contains several antinutritive nutrients (e.g., tyrosine inhibitors, saponins, phytic acid, lectins, etc.). However, its amphipathic property makes it an excellent emulsion for the production of aqueous extracts [22].

Based on their main ingredient, milk alternatives can basically be classified into five main groups: cereals (e.g., oats, rice), legumes (e.g., chickpeas, soybeans), nuts (e.g., almonds, Brazil nuts, cashew nuts, hazelnuts), seeds (e.g., sesame, sunflower), and pseudo-grains (e.g., quinoa) [16,20].

Pseudocereals, such as quinoa, buckwheat, and amaranth, are also playing a prominent role in the development of plant-based dairy substitutes. Due to their high protein content, superior nutritional value, and low cost, quinoa proteins, for example, have been identified as a viable alternative for the production of plant-based dairy products [20].

Although milk and dairy products are of high biological value, containing complete nutrients, vitamins, and minerals (calcium, magnesium, and phosphorus) that are essential for maintaining a healthy body, their consumption has been declining recently. This is particularly true in Western societies and is mainly due to the fact that milk is fundamentally more difficult to digest, while several sources have shown that metabolic diseases, lactose intolerance, and milk protein allergies have a high incidence in the population. According to some studies, nearly three quarters of the world’s population suffer from some form of lactose intolerance (e.g., diarrhea, abdominal distension, constipation, and abdominal discomfort) [22]

Recently, there has been an increased demand for plant-based milk alternatives to avoid these problems. A major advantage of plant-based milk alternatives is that they resemble bovine milk in appearance and often in taste and can, therefore, be used as a functional substitutes for milk [20].

These drinks do not contain cholesterol and lactose, but they may contain allergens and are low in protein, so they cannot replace the nutritional value of milk [22]. The consumption of plant-based beverages among people with food allergies, intolerances (especially lactose intolerance), and vegan or vegetarian diets has been significant over the last decade. They are also widespread among people suffering from celiac disease, as many drinks are made of gluten-free cereals or pseudo-grains, pulses, oilseeds, or nuts. The protein content of plant-based drinks has been found to range from 0.06% (cereals) to 4.3% (pulses), but when supplemented with other ingredients (e.g., plant protein sources) or when the raw materials are combined, the protein profile can be improved [23].

**Figure 2:**
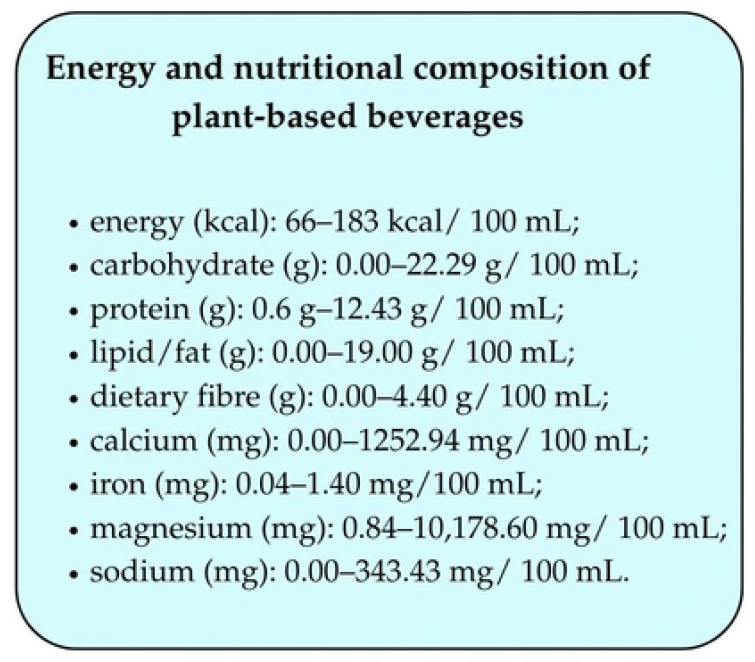
Energy and nutritional composition of plant-based beverages.

The energy and nutritional composition of plant-based beverages is shown in Figure 2 [24].

Plant-based milk alternatives have the advantage of containing naturally occurring bioactive substances, as follows [24]:

• Soy-based beverages—isoflavones, phytosterols;
• Almond-based beverages—α-tocopherol, arabinose;
• Oat-based beverages—ß-glucans.

#### 5.1.4. Pre- and Probiotic Drinks

Prebiotics found in these drinks include milk oligosaccharides, fructooligosaccharides (FOS) and glucooligosaccharides (GOS, maltodextrin), while probiotics include *Lactobacillus reuteri*, *Lactobacillus acidophilus*, and *Bifidobacterium* spp. [3].

#### 5.1.5. Sports Drinks

Sports drinks are recommended to be consumed before or during exercise, primarily for the purpose of improving performance, concentration, and endurance [15]. These drinks are carbohydrate–electrolyte beverages e.g., isotonic, hypotonic, hypertonic drinks to hydrate the body. They usually contain caffeine (or other methylxanthines), flavonoids, e.g., catechin, ginseng, vitamins B, guarana, acai, ginkgo, inositol, yerba mate, carnitine, taurine, glucuronolactone, or creatine [3].

#### 5.1.6. Energy Drinks

Energy drinks are blends of stimulants and energy boosters that improve cognitive function, reduce fatigue, improve stamina, and maintain alertness. Their main ingredients are sugars or artificial sweeteners, amino acids, taurine, caffeine, vitamins (vitamin B6, niacin, vitamin B12, vitamin C, and vitamin B5), and minerals (sodium, calcium, and magnesium), but they may also contain guarana, ginseng, yerba mate, and ginkgo [3,25]. Studies have also highlighted the harmful effects of frequent consumption of energy drinks (e.g., ischemic effects, nervous system problems, insomnia, etc.) [3]. Some of the raw materials (e.g., high sugar, especially fructose content, taurine, and carbonic acid) [15] often cause lipogenesis, which can lead to non-alcoholic fatty liver and insulin resistance [3].

#### 5.1.7. Phytoactive-Fortified Drinks

These drinks are fortified with soy-based bioactive components, such as phytosterols and isoflavones, bioactive almond extracts containing arabinose and α-tocopherol, oat extracts containing β-glucan, and sea buckthorn extract containing β-sitosterol and vitamins [3].

#### 5.1.8. Beauty Drinks

The most important functional ingredients of beauty drinks are collagen (bovine collagen or fish collagen), hyaluronic acid, calcium lactate pentahydrate, red clover (*Trifolium pratense*), fucoxanthin, theobromine, oryzanol, eicosapentaenoic acid (EPA), and amino acids to increase skin hydration [3].

#### 5.1.9. Cognitive- and Immune-Enhancing Drinks

These are beverages enriched with probiotics that, when consumed in sufficient quantities, produce neuroactive compounds, such as gamma-aminobutyric acid (GABA), noradrenaline, dopamine, and serotonin. These have been found to regulate brain-derived neurotrophic factor (BDNF), which is important for central nervous system-related behaviors and is linked to gut–brain communication [3].

Functional drinks include yoghurt drinks fortified with pre- and probiotics, milk fortified with calcium, as well as possibly omega-3 fatty acids and vitamins (vitamin D), (“functional milk”), fruit and vegetable juices fortified with omega-3 fatty acids, iron, and vitamins, and “functional waters” fortified with vitamins and minerals (e.g., magnesium) and possibly considered as sports and energy drinks, as well as herbal drinks (e.g., aloe vera) and health and wellness drinks. According to a 2019 study, these are the beverages with the highest market penetration [3,26].

### 5.2. Types of Functional Beverages According to Their Condition-Specific Properties

There are also other classifications of functional beverages. According to their impact on health or their condition-specific properties, we can distinguish eight types of functional beverages, as follows [3]:

1. Energy—vitamin C, tannins, caffeine;
2. Performance—electrolytes, ginseng, proteins;
3. Nutricosmetics—trans-resveratrol, ß-sitosterol, anthocyanidins;
4. Cardiovascular—phytosterols, isoflavones, fibers;
5. Cognitive health—citicoline, selenium, resveratrol;
6. Immunity—eugenol, apigenin, ascorbic acid;
7. Digestive health—fibers, enzymes, lactic acid;
8. Weight management—capsaicin, curcumin, flavonoids.

### 5.3. Classification of Functional Ingredients Used in Functional Drinks

Functional drink ingredients include a very wide range of food components and biologically active substances, such as fruits (e.g., apples, bananas, oranges), tea, coffee, cocoa, soybeans, dried plants, animal products (e.g., dairy products, eggs, fish oil), herbs and spices, added proteins [9], minerals or vitamins (e.g., calcium-fortified orange juice, folic acid-fortified bread, iron-fortified fruit juice), probiotics, prebiotics, omega-3 fatty acids, and antioxidants (e.g., flavonoids) [13]. Raw materials, such as water, plant-based milk alternatives, fruit juices, milk, herbal teas, and other microbial substrates, are used as bases [3].

The literature classifies functional ingredients into the following groups. Table 1 shows the components of functional beverages.

### 5.4. Biologically Active Ingredients of Functional Beverages

Functional drinks have received so much attention in the first place because of the biologically active ingredients that they contain. These ingredients play an explicit role in enhancing the health of the body [3]. In addition to containing bioactive components from natural sources, functional beverages may also contain bioactive components added separately to these products. It is, therefore, essential to highlight the bioactive ingredients separately.

These bioactive compounds are mostly derived from animal products, herbs, spices, seafood, and microorganisms [3]. Such ingredients include antioxidants, including omega-3 fatty acids, polyphenols, phenolic compounds, phenolic acids (e.g., hydroxycinnamic acid, chlorogenic acid, caffeic acid, etc.), carotenoids, flavonoids, tannins, catechins, quercetin, kaempferol, rutin, luteoyl, naringin, terpenes, etc. Dietary fibers, prebiotics, probiotics, certain bioactive proteins, peptides, unsaturated fatty acids, minerals (e.g., magnesium, phosphorus, folic acid, and zinc) and vitamins (e.g., vitamins A, C, and D) are also considered as biologically active ingredients used in the development of functional drinks. Evidence shows that added fiber, vitamins, and minerals in functional beverages play a potential role in reducing diseases [3,9]. Some research suggests that cannabidiol (CBD), nootropic, allergen-free plant protein sources, and adaptogen ingredients will be particularly important in functional drinks in the near future [3,59,60].

#### 5.4.1. Antioxidants

Antioxidants are functional compounds that are able to neutralize harmful free radicals, thereby reducing the occurrence of oxidative stress and inflammatory processes, and, thus, reducing the risk of certain chronic diseases (e.g., cardiovascular diseases and cancers). Foods with a high antioxidant activity (in particular certain fruits, vegetables, nuts, and oilseeds) play an important role in preventing the development of oxidative stress caused by free radicals [1]. Natural sources of antioxidants include fish oil (EPA, DHA), walnuts, and flaxseed (ALA), but they also occur in vegetables, fruits (vitamin C, ß-carotene, lycopene, chlorophyll or anthocyanins, selenium), cocoa (theobromine), coffee (quercetin, chlorogenic acid), and tea (EGCG), as shown in Table 1.

Phenolic compounds are generally phytochemicals that are found in all plants. Their role is primarily in the prevention of oxidative stress, and they are most commonly found in fruits and vegetables, but can also be found in cereals, soy, tea, and coffee beans [9].

It is important to note that when certain foods with high antioxidant activity are combined with each other, a synergistic effect is observed. A good example of this is the research by Makanjuola and colleagues [40] who combined green tea with a ginger mixture and found that the resulting drink had better antioxidant properties, probably due to the increase in polyphenols [9].

#### 5.4.2. Omega-3 Fatty Acids

Omega-3 fatty acids are considered to be antioxidants, with potential benefits primarily in cardiovascular disease due to their ability to improve and regulate lipid levels and reduce the risk of heart disease [61,62]. Based on studies, omega-3 fatty acids are essential components of functional foods [1].

Omega-3 fatty acids are mainly found in some plants (e.g., flaxseed, rapeseed, and walnuts (alpha-linolenic acid, ALA)) and fatty fish e.g., tuna, salmon, and mackerel (eicosapentaenoic acid, EPA and docosahexaenoic acid, DHA). ALA is essential, whereas EPA and DHA are converted from ALA by the body. However, it is important to introduce these from external sources as well in order to cover the body’s needs as much as possible [1]. Thus, omega-3 fatty acid-rich oils extracted from fish are used in functional drinks, and the use of seaweeds has recently become common alongside fish. In addition to natural sources, omega-3 fatty acid-rich non-probiotic milk-based drinks are also available on the shelves [3].

In addition to their positive effects on the cardiovascular system, omega-3 fatty acids are also important for the development of the immune system, the central nervous system, and the eyes. For example, DHA is a major component of the brain structure, and research shows that its formation and intake from external sources is associated with increased cognitive performance. In addition, omega-3 fatty acid intake may improve memory and cognitive decline with aging, as well as reducing the risk of Alzheimer’s disease [1].

#### 5.4.3. Prebiotics and Probiotics

With the increased research on the gut microbiome, the focus on the balance of the gut microbiota has increased in recent decades. This is why the last decade has seen an increase in research on the use of pre- and probiotics in functional food production to promote health [16] and an increasing role for prebiotics and probiotics in diets in product and health promotion [1]. Several studies on the gut microbiome have investigated the relationship between gut microbiome health and mental health, having shown that the greater the diversity of the gut microbiome is, the more favorable its cognitive effects are [1,63].

Prebiotics are specific fermentable components that modify the function or composition of the gut microbiota to the benefit of the host [13]. Prebiotics are nutrients for beneficial bacterial strains of the gut microbiota (Lactobacilli, Bifidobacteria), thereby enhancing their proliferation and increasing the diversity of the gut microbiota [1,13]. It has been shown that during fermentation, beneficial bacteria produce short chain fatty acids (SCFAs) and reduce pH, thus providing an unfavorable environment for harmful bacteria and also protecting the intestinal mucosa [13]. The ten most abundant prebiotics are found in Table 2 [13].

Excellent sources of prebiotics include onions, garlic, Jerusalem artichokes, bananas, and whole grains, including oats and barley, which contain mainly fructo-oligosaccharides (e.g., bananas), inulin (e.g., onions, Jerusalem artichokes), or ß-glucan (e.g., oats, barley) [1]. Some seaweeds also have prebiotic properties [3].

Among the prebiotics, galacto-oligosaccharides enhance Lactobacillus, while the combination of galacto-oligosaccharides with fructo-oligosaccharides increases the proportion of Bifidobacteria and reduces the proportion of harmful bacteria (e.g., Clostridium), thus preventing and treating many chronic diseases. In addition, inulin, pectin, and arabino-xylo-oligosaccharides improve intestinal barrier function and immune response [13]. Resistant starch is also considered a prebiotic, and research suggests that it may reduce the risk of colon cancer and increase dietary magnesium and calcium intake. Polyphenol–starch complexes can be used to develop potentially healthier functional foods [64]. It was observed that supplementation with prebiotics, especially maltodextrin, pectin, mannitol, and inulin, improved the viability and reproduction of probiotics (*Lactobacillus reuteri*, *Lactobacillus acidophilus*, and *Bifidobacterium* spp.) [3].

Functionally beneficial fruits include citrus fruits, such as oranges, lemons, limes, and grapefruit, red berries, such as raspberries, blueberries, blackberries, blackcurrants, redcurrants, strawberries, and red grapes [1]. Kiwi, acai, cherry, peach, pomegranate, grape, and strawberry have perishable surfaces, with the positive properties of being fermentable by *Leuconostoc*, *Lactobacillus*, and *Enterococcus* species, thus producing probiotic juices and fruit preparations [3].

Table 2 shows the different types of prebiotics and prebiotic sources.

**Table 2:** Prebiotics and prebiotic sources.

Prebiotics	Prebiotic Sources	References
Inulin	Jerusalem artichoke (tubers), chicory, onion, garlic, barley, dahlia	[65]
Lactulose	Heat-treated milk	[54]
Xylo-oligosaccharides	Leeks, asparagus	[66]
Resistant starch	Cooked and cooled high amylose content foods (rice, sweet potato, wheat, pulses, lentils, beans, etc.)	[67]
Pectin	Orange, apple	[68]
Arabinoxylan	Whole grains of cereals and pseudocereals	[69]
Galacto-oligosaccharides	Coffee, dairy products	[70]
Fructo-oligosaccharides	Artichokes, asparagus, bananas, chicory root, garlic, onions, leeks, and wheat	[71]
Mannan saccharides	Konjac and the outer cell wall membrane of bacteria, plants, or yeast	[72]
Beta-glucans	Barley, oats, bacteria, yeast	[66,73]

Probiotic-based functional drinks are widely known. In fact, when consumed in adequate quantities, these products can lead to several nutritional benefits, such as boosting immunity, reducing serum cholesterol levels, improving lactose intolerance, alleviating inflammable bowel diseases, improving digestive health, safeguarding against infection, and reducing the risk of developing cancer, cardiovascular diseases, diabetes, allergies, and intolerances [2].

Commonly used probiotic strains in functional beverages include the following [2]:

• Lactobacillus—*L. rhamnosus*, *L. acidophilus*, *L. plantarum*, *L. casei*, *L. brevis*, *L. reuteri*.
• Bifidobacterium—*B. infantis*, *B. animalis subsp.lactis*, *B. bifidum*, *B. longum*, *B. breve*.
• Saccharomyces—*S. boulardii*.
• Escherichia—*E. coli Nissle 191*.
• Bacillus—*B. coagulans*, *B. subtilis*, *B. cereus*.
• Streptococcus—*S. thermophilus*.
• Enterococcus—*E. durans*, *E. faecium*.

Foods rich in probiotics include kimchi in Korea, natto in Japan, miso in Japan and in Korea, tempeh in Indonesia, kombucha in China, mabundu in Tanzania, sourdough bread in Europe and in the USA, and sauerkraut in Europe [2]. Other probiotic foods include probiotic yoghurts, cheese, tofu, and soured/cultured milk (Lactobacillus) [1,2].

Fruit- and/or vegetable-based functional drinks are usually produced by fermentation or fortification. Fermented functional fruit drinks have microorganisms added, e.g., *Lactobacillus* spp., *Lactobacillus rhamnosus*, and *Saccharomyces cerevisiae* [3,33].

Oats are often used in the development of functional drinks, as they ferment well with, for example, *Lactobacillus plantarum* starter culture, without reducing the ß-glucan levels of oats when properly dosed [3,34].

#### 5.4.4. Bioactive Peptides and Proteins

Bioactive peptides are chains of amino acids released during gastrointestinal digestion or food processing that have antioxidant, antimicrobial, antitumor, immune-boosting, blood pressure-lowering, and even cholesterol-lowering properties. Their role is primarily in metabolic regulation, which is why they are often used as an ingredient in functional foods. Peptides with biological activity occur naturally in fermented dairy products (e.g., yoghurt, cheese) [9]. In addition to these sources, bioactive peptides can also be obtained from seafood, such as fish protein hydrolysates and amino acid peptide chains [3].

Common ingredients in functional drinks are milk proteins, including whey proteins, which are mostly added to protein preparations, and casein, which is an important precursor of biologically active peptides. These peptides can generate compounds that are useful in inhibiting angiotensin-converting enzymes [3].

In a study, lyophilized ovalbumin (a protein extracted from egg whites) was added to a functional drink produced by enzymatic hydrolysis. By this method, the solubility of the protein was more favorable than that of the native protein beverage, and its antioxidant, antimicrobial, and metal-chelating activities were enhanced. The study found that a functional drink made with hydrolyzed ovalbumin can be stored for up to one week at 4 °C [74].

#### 5.4.5. Vitamins and Minerals

As mentioned above, the main ingredients added to functional drinks are iron, calcium, magnesium, phosphorus, and zinc. Among vitamins, vitamins A, D, E, K, and C and B-complexes (e.g., B1, B2, B3, folic acid, B12) are significant [3].

In most functional beverages, it is important to add natural vitamin and mineral sources in order to reach the target vitamin and mineral levels. Non-probiotic fruit and vegetable drinks are produced by adding vitamins and minerals (e.g., iron-fortified juices). Milk-based drinks are also often fortified with vitamin D and minerals (e.g., calcium or magnesium) to compensate for the loss of nutrients during milk processing [3].

Water is one of the most important natural source of bioactive compounds (e.g., calcium) in functional drinks, and the choice of water should not be overlooked, as some of the ingredients in water can affect the functional properties of the drink. A good example is that the hardness of water affects the pH, which can lead to a change in taste or even in the proportion of microbial species [3].

Table 3 shows the natural sources of the mentioned minerals and vitamins [75].

#### 5.4.6. Dietary Fiber

In addition to prebiotics and prebiotic fiber, dietary fiber should also be mentioned, which is also present in functional drinks, either added separately or from natural sources. The health links between regular fiber intake and metabolic status have been already shown. Fiber intake increases the diversity of the gut microbiota, thereby improving metabolic function, reducing insulin resistance, and even preventing obesity. This is due to the property that after its intake, the fiber in the large intestine is fermented by intestinal bacteria, which produces short-chain fatty acids. In addition, they induce the production of metabolites, such as phenolic and indole compounds, branched chain fatty acids, lactate, succinate, and various gases (hydrogen, carbon dioxide, methane, nitric oxide, and sulfur compounds). However, the “precursors” used to produce these metabolites are dependent on dietary intake, thus suggesting that fiber intake is a key factor in the prevention and treatment of many metabolic diseases [76].

Fibers belong to the group of carbohydrates, formally edible but indigestible parts of plants, chemically classified according to molecular weight (polysaccharides, consisting of at least 10 monomers). Dietary fiber is defined by the European Food Safety Authority (EFSA) as non-digestible carbohydrates and lignin, which may be composed of substances, such as non-starch polysaccharides, cellulose, pectins, hydrocolloids, fructo-oligosaccharides, or “resistant starch” [77].

The main benefits of dietary fiber include the following [77]:

• Improving gut motility.
• Reducing body weight and abdominal adiposity.
• Improving insulin sensitivity and metabolic health.
• Increasing the diversity of gut microflora and metabolites.
• Preventing and alleviating chronic inflammation.
• Preventing and treating depression.
• Preventing and treating cardiovascular disease.
• Preventing colorectal carcinoma (crc).
• Reducing mortality.

There are two broad groups of fibers, namely water-soluble and water-insoluble dietary fibers. The primary sources of water-soluble fiber are fruits and vegetables (e.g., inulin, pectin), while the water-insoluble types are cereals and pseudocereals (e.g., cellulose, lignin) [77,78]. One of the main roles of water-soluble dietary fiber is to increase the viscosity of the intestinal contents, while non-water-soluble dietary fiber binds water, thereby loosening the stool structure and increasing its bulk [69]. Several dietary fibers are considered to be prebiotics, as shown in Table 3 [77].

The physiological functions of dietary fibers are shown in Table 4.

#### 5.4.7. Sweetening Components

The expectations towards functional beverages require that they are naturally sweetened, i.e., they do not contain added sugar or artificial sweeteners [3]. In addition, consumers’ choice of product is influenced to a large extent when they see “WITH NO ADDED SUGAR” and “CONTAINS NATURALLY OCCURRING SUGARS” among the mandatory information on the label. In the European Union, mandatory labeling claims are intended to inform consumers about food properties according to REGULATION (EC) No 1924/2006 [80,81].

According to other studies on functional beverages, aspartame, acesulfame K, sucralose, and stevia are commonly used sweeteners [58].

Interestingly, the use of *Lactobacillus reuteri*, *Lactobacillus acidophilus*, *Bifidobacterium* spp., and other microbial species produces mannitol as a prebiotic during the fermentation process. This increases the viscosity and antioxidant and antibiotic properties of the drink, giving it a sweeter taste [3]. A well-known prebiotic, lactulose, is also a favorable ingredient in functional beverages due to its technological properties. The sweetness of lactulose is 48–62% of that of sucrose, with no aftertaste. It has high solubility, as well as low cariogenic potential and high stability [54].

The date (*Phoenix dactylifera* L.) can be a suitable sweetener for functional drinks, as the fresh fruit has a high natural sugar content (80 g/100 g). In addition to being a sweetener, it also contains cellulose, pectin, vitamins, antioxidants, and phenolic compounds. It can be used as fresh fruit or as extracted date syrup in functional drinks [55].

### 5.5. Processing of Functional Beverages

The production of plant-based beverages is not easy, as these plant-based ingredients have unfavorable side tastes (e.g., bitter, metallic, sour, grassy, etc.) and are often discolored due to enzymatic browning and sediment formation. These properties may be due to the presence of fats, proteins, and starch granules in the base material in plant-based beverages [16]. These plant-based functional drinks are produced by the aqueous extraction of plant ingredients (e.g., pulses, fruits, cereals, nuts, seeds, and leaves). The technologies used include maceration, grinding, and filtration [16].

In addition to producing a functional drink, attention to shelf life and storage conditions is also an important aspect in order to preserve the storage stability of the product, which will be an important determinant of its quality. Among the preservation processes, heat treatment is an essential process that helps to maintain microbial stability, eliminate undesirable flavors, odors, volatiles, and antinutrients, and enhance the extraction process [79]. Several methods are available for processing functional drinks, including both heat and non-heat-treatment techniques [3].

#### 5.5.1. Heat-Treatment Processes

The primary objective of heat-treatment processes is to increase the shelf life of the heat-treated product, i.e., to inactivate pathogenic microbes and spoilage enzymes [3]. Heat-treatment processes may also result in the formation of undesirable substances (e.g., acrylamide, esters, etc.) or the degradation of certain heat-sensitive substances (e.g., vitamin C). Today, advanced technologies, such as ultra-high temperature processing, high temperature short-time processing, ohmic heating, and electromagnetic heating are used to preserve functional beverages by heat treatment [82]. Traditional heat treatment processes can be divided into several categories, namely short-time mild temperature treatment (MTST, <80 °C ≤30 s), long-time mild temperature treatment (MTLT, <80 °C >30 s), short-time high temperature treatment (HTST, ≤80 °C to <30 °C between 30 s), long-time high temperature treatment (HTLT, ≤80 °C to <130 °C between >30 s), and ultra-high temperature treatment (UHT, 130 to 150 °C between 2 to 10 s). Among the non-traditional, novel processes, ohmic heating (OH) and microwave heating (MH) have been shown to be less destructive to the food matrix [3].

In the case of chilled beverages, pasteurization is the preferred preservation method, whereas for non-chilled beverages, packaging methods and other more efficient methods help to achieve microbial stability and preserve product quality. Depending on the packaging, this allows the beverage to keep for several months, whereas in the chilled format it can only be consumed for a few days. At the same time, studies have shown that storage temperature, exposure to light, or the oxygen permeability of the packaging can affect the contents of vitamins, minerals, and other bioactive compounds, as well as the stability of the product [82].

#### 5.5.2. Non-Heat Treatment Processes

Non-temperature treatments of functional drinks are used to improve the preservation of the food matrix and are, therefore, becoming increasingly common. Examples of such processes include high-pressure processing (HPP), pulsed electric field (PEF), supercritical carbon dioxide (SC-CO_2_) technology, ultrasonic processing (US), cold plasma processing (CPP), and membrane technology (MT). Ultrasonic processing, for example, produces sound waves that generate free radicals and micromechanical shocks to combat the microorganisms [3]. Ultrasonic waves with high intensities (10–1000 W/cm^2^) and low frequencies (20–100 kHz) used in food processing extraction processes (e.g., protein extraction of soy milk) have significant physical and chemical effects [82]. Atmospheric cold plasma (ACP) processing has a stimulatory effect on bioactive compounds in functional beverages [3].

Microwave extraction (MAE) achieves cell wall disruption by forced superheating of water molecules and continuous collisions within the matrix. As a consequence of this process, the components inside the cells are rapidly released into the surrounding solvent [82].

## 6. The Importance of Plant-Based Protein Intake

The explosion in population growth has also made it necessary to meet the growing demand for protein, and an effective solution is to increase the intake of plant-based proteins in the diet, with pseudo-grain products being excellent examples, alongside pulses and cereals. In addition to the protein content, it is also important to consider the biological value, digestibility, and amino acid composition of the plant protein source. Another important aspect is the essential amino acid content, which cannot be synthesized by the body and, therefore, needs to be obtained from dietary sources. Plant-based protein sources often do not contain sufficient essential amino acids (around 90%) and also have a lower amino acid score (AAS); therefore, they considered to be “incomplete” proteins [11]. The digestibility and absorption of hydrolyzed protein products in the human gastrointestinal tract is the most important indicator for assessing the quality of proteins, in addition to the amino acid composition [83].

The ratio is shifted by the presence of the limiting amino acid. The limiting amino acid is the amino acid that is present in the smallest proportion in a given protein, thus limiting the utilization of the other essential amino acids. For cereals, this amino acid is lysine. However, regular intake of plant-based protein sources increases essential amino acid intake, and different plant protein sources can complement each other’s amino acid profile on average over a day (e.g., if cereals, legumes, oilseeds, etc. are consumed during the day). Regular intake of diverse plant-based protein sources is also essential to meet essential amino acid requirements. The presence of antinutritional compounds or the absorption process and digestion conditions make the digestion of proteins more difficult. For example, the availability of lysine, the limiting amino acid in cereals, may be reduced by Maillard reactions during heat treatment, which has a marked effect on the synthesis of proteins in the body. PER, PDCAAS, and DIAAS are relevant methods that are able to determine the quality and digestibility of proteins [11].

In a study, protein digestibility was determined in vitro as the ratio of the nitrogen content of the digested portion (Nd) to the nitrogen content of the whole beverage before digestion (Ns), which may also help to determine the protein content of other beverages. As a correction, the nitrogen content of the enzyme was used in a blank test (Nb). The Kjeldahl method was used to determine the nitrogen content of the sample, and the protein digestibility of each sample was determined according to the following equation [83]:

Protein Digestibility (%) = (Nd − Nb)/Ns × 100.

### 6.1. Protein Efficiency Ratio (PER)

The protein efficiency ratio (PER) is considered as an initial method of assessing the protein quality of foods. In some countries (e.g., Canada), the PER is used to determine the protein classification of a food with a given protein content. The method is not accurate and, therefore, the PER value is determined using the protein digestibility-corrected amino acid score (PDCAAS) method (PDCAAS × 2.5 = estimated PER). This change was justified by the recognized limitations of PER [11].

### 6.2. Protein Digestibility Corrected Amino Acid Score (PDCAAS)

The use of the PDCAAS method is widely recognized for the assessment of protein quality [12]. This method provides an accurate estimate of protein values considering the digestibility and essential amino acid profile of proteins [11]. It compares the balanced ratio of essential amino acids and the degree of digestibility to a value of 1.0, which is the value of milk. Furthermore, 1.0 is the maximum PDCAAS value [83].

### 6.3. Digestible Indispensable Amino Acid Score (DIAAS)

In 2013, the FAO/WHO recommended the use of the DIAAS method to address the limitations of the PDCAAS. The DIAAS method is similar to the PDCAAS method with some important differences [12]. DIAAS proves to be superior to PDCAAS because it measures the true ileal digestibility of proteins and not the digestibility determined from feces [83]. An ileal digestibility coefficient is assigned to each essential amino acid in the protein source. It is often used for in vivo characterization of food proteins [12]. The amino acid score is adjusted accordingly and, thus, becomes comparable to the scoring sample conditions (mg/g) per age group. Unlike PDCAAS, scores higher than 100% can be determined with the DIAAS method [11].

## 7. Pseudocereal-Based Functional Beverages

In recent years, global production of rice, wheat, and maize has increased dramatically, tripling according to some sources. According to a 2019 study, increased production has led to increased availability and consumption, accounting for nearly half of total energy intake in recent times [84]. The high consumption of these nutrient-poor cereals has contributed to obesity, cardiovascular disease, insulin resistance, etc., and, in addition, diets have been deficient in dietary fiber, high-quality protein sources, minerals (e.g., iron, zinc, calcium), and bioactive compounds (e.g., polyphenols). According to FAO research, around 1.3 billion people worldwide suffer from micronutrient deficiencies [10].

Avoiding nutrient deficiencies demands a diet rich in micronutrients, containing good quality ingredients and a variety of foods [10]. Recently, there has been an increased interest in the use and incorporation of pseudocereals into diets, as they have a better nutritional composition than some cereals (e.g., rice, maize). In addition to their higher amino acid value, they have a better protein composition and a higher resistant starch content and, thus, a lower glycemic index (GI) than cereals. In addition, they contain a number of biologically active substances [85]. To this end, it would be advisable to reduce the frequent use of rice, maize, and wheat, and to increase the use of pseudocereals.

Pseudocereals have specific functional properties, they are highly genetically variable and well adapted to different climates, making them easily cultivated in both temperate and tropical climates and, therefore, increasingly common crops worldwide [12].

Unlike cereals, pseudocereals are not members of the grass family *Poaceae* [86]. Pseudocereals are dicotyledonous plants, while cereals (e.g., wheat, rice, and barley) are monocotyledonous [69]. Along with this, they are edible seeds that resemble cereal grains in both appearance and properties [86], and can, therefore, be used in a similar way to cereals [12].

Recent research has shown that the functional diversity of plant-based functional drinks depends not only on the added biologically active substances they contain, but also on the type of substrate. This is why the use of pseudocereals in functional drinks has become increasingly popular, as they have a favorable protein composition (rich in essential amino acids), a high dietary fiber content, high levels of vitamins C, E, and B, a low starch content, but a higher protein content than cereals. They are also gluten-free [16].

There are three common pseudocereals, namely quinoa, amaranth, and buckwheat (common buckwheat or Tatar buckwheat) [10,12]. Quinoa consumption has beneficial effects on digestive, metabolic, and cardiovascular functions [29]. It was observed that the consumption of buckwheat had a positive effect on the lipid profile of the blood, reducing levels of total cholesterol, triglycerides, and low-density lipoproteins (LDL cholesterol) and increasing levels of high-density lipoproteins (HDL cholesterol). As a result of its regular consumption, the incidence of type 2 diabetes, cancer, and cardiovascular disease was reduced [35].

### 7.1. The Structure of Pseudocereals

Like cereal grains, the seeds of pseudocereals are made up of three main parts. From the outside inwards, the first part is the hull layer, which is high in fiber and color, but also protective against external aggressions. The next part is the inner core, which carries the nutrient content and is rich in complex carbohydrates and vegetable proteins. The last and innermost part is the germ layer, which contains unsaturated fatty acids and vitamins and ensures the development of the plant [86]. Pseudo-grains are processed into flour and used in bakery products and beverages, as well as in milk and dairy alternatives [85].

The diameter of the amaranth seed is about 0.9 to 1.7 mm. The largest part of the seed is the embryo, which consists of root hairs and petals. The embryo, the perisperm, and the petiole are the main protein reservoirs of the seed. The seed coat has a smooth surface and can be white, cream, golden yellow, or even brown in color. Quinoa seeds are similar in structure, but slightly larger in diameter, ranging from 1.0 to 2.6 mm. The seeds are small and spherical, with the petiole forming the main protein storage tissue. The buckwheat consists of characteristic pyramidal seeds, ranging from 4 to 9 mm in diameter. The seeds are brown or grey in color and covered by a so-called pericarp. The storage tissues are similar to those of amaranth and quinoa [69].

### 7.2. Types of Pseudocereals

Among the pseudo-grains, three main species can be distinguished, namely quinoa (*Chenopodium quinoa Willd*), amaranth (*Amaranthus* sp.), and buckwheat (*Fagopyrum* sp.), of which two species can be distinguished, namely the common buckwheat (*Fagopyrum esculentum Moench*) and the Tatar buckwheat (*Fagopyrum tataricum Gaertn*). Quinoa and amaranth belong to the family *Chenopodiaceae* and are native to the Andean regions of South America. Buckwheat, of the family *Polygonaceae*, is native to central and western China [10,12].

Pseudo-grains do not contain gluten-containing proteins and are, therefore, a preferred ingredient in gluten-free breads and bakery products [85]. The consumption of pseudocereals is gaining ground as gluten-free alternatives for those with celiac disease or non-celiac gluten-sensitive enteropathy (NCGS). They are also versatile and have excellent nutritional value [12].

#### 7.2.1. Quinoa

The edible parts of quinoa are its green leaves, which are high in chlorophyll, and its edible seeds [87]. Quinoa seeds are classified according to color, size, and texture, but are most often distinguished by their color [88]. The seeds are round and flat, with diameters ranging from 1.5 mm to 4 mm, and come in different sizes and colors [87]. In terms of color, they can be distinguished as red, white, or black [88]. Overall, quinoa has a better nutritional value than other pseudo-grains or cereals [88], as it has a balanced protein, carbohydrate, and lipid composition. Furthermore, 2013 was the FAO’s “Year of Quinoa” [87]. It is interesting to note that quinoa is the only pseudo-legume and the only crop that provides all the essential nutrients required by humans, according to the Food and Agriculture Organization (FAO). In addition to being a healthy and nutritious food, it is also considered by the FAO to be the “smart food of the future” [20], because it retains its micronutrient content in extreme climatic conditions and requires little energy to produce [87].

According to some sources, it is a pseudo-grain from the Andes, Peru, and Bolivia. Its health benefits include antioxidant, anti-obesity, and hypocholesterolemic effects. In particular, high antioxidant activity has been demonstrated in darker quinoa seeds. Phytosterols in quinoa are involved in lowering serum cholesterol level [36].

It is high in vitamin B (especially in vitamins B1, B2, and B3) and has been shown to have anticarcinogenic and cardiovascular protective effects. The red and black seeds are rich in flavonoids (e.g., ferulic acid, quercetin, kaempferol, rutin, etc.) [36].

The use of quinoa grains is similar to that of rice, as they are used in cassava, muesli, as flour, cooked as a side dish, or added to extruded products [89].

#### 7.2.2. Buckwheat

Buckwheat species (common or Tatar) are well adapted to environmental and climatic changes and are, therefore, widely cultivated [90]. They are annual biennial plants with triangular brown thistle fruits. The buckwheat fruit is brown or purplish–black, measuring about 4–7 × 3–4 mm [85]. The seeds are brown or grey and range from 4 to 9 mm in diameter [69]. Buckwheat is also very rich in bioactive compounds, thus making it a pseudo-grain particularly suitable for human consumption [90]. The seeds of buckwheat have many health and nutritional benefits [36]. Buckwheat is most often processed into flour and is, therefore, a popular ingredient in breads, pastries, biscuits, beer, wine, and vinegar [90]. The resistant starch content in the raw seed is one-third of the total starch content, while after cooking it is about 7–10% [85]. Buckwheat is seen as an underutilized, “future-smart” crop with the potential to contribute to global food and nutrition security. In vivo and in vitro studies on buckwheat have shown its anti-diabetic, anti-tumor, anti-inflammatory, antioxidant, and hepatoprotective effects, and its consumption can prevent hypertension, obesity, cardiovascular diseases, gallstone formation, cancer, and many non-communicable diseases [90].

As with quinoa and amaranth, buckwheat is gluten-free and contains bioactive compounds, such as rutin, quercetin, orientin, isoorientin, and isovitexin. Interestingly, some studies have shown a higher antioxidant activity in buckwheat groats compared to common buckwheat [36].

#### 7.2.3. Amaranth

Amaranth is also a member of the pseudocereal family [91]. There are 70 species in the genus *Amaranthus*, but only a few species are a source of nutrients for humans. The most common species are *Amaranthus caudatus* (Peru), *Amaranthus cruentus* (Guatemala), and *Amaranthus hypochondriacus* (Mexico) [92]. The plant itself is annual, about 0.9–1.8 m tall, and was a staple food in the Incan, Mayan, and Aztec civilizations. Its seeds are used mainly as a source of flour and oil. It is an important ingredient in gluten-free breads, and other parts of the plant are added to salads or soups [91].

Both the seeds and the leaves have a higher nutritional value than the cereal components. Its chemical composition depends on its species, variety, and climatic conditions [91]. In terms of nutrient composition, amaranth has a significant content of protein (11.7–22%), starch (65–75%), unsaturated fatty acids, dietary fiber, vitamins, and minerals (about 3.3% of dry matter [12,37,92,93,94]. Another source indicates that the starch content is about 45–65%. The fiber content is about 2–8% of the dry matter, with the water-soluble part being pectin and the water-insoluble part comprising lignin, cellulose, and hemicellulose (e.g., xyloglucan [37,38]. Some sources indicate that the fiber content may be as high as 8–16% [32].

It also has a high unsaturated fatty acid content (about 75% in 6–9% oil [61.0–87.3%]) [10,12,91]. The unsaturated fatty acids are mainly linoleic acid (about 62%), oleic acid (about 20%), alpha-linolenic acid (about 1%), and arachidonic acid. It also contains tocopherols, tocotrienols, and sterols [37].

It is high in calcium, potassium, magnesium, iron, phosphorus, manganese, zinc, and copper [37,38], and also contains polyphenols, saponins, hemagglutinins, phytin, nitrates, and oxalates. Among its bioactive compounds, betacyanins, including betanidine [37], and rutin, isoquercetin, and nicotiflorin, are prominent [38].

### 7.3. Nutrient Content of Pseudocereals

In 2011, the World Food and Agriculture Organization (FAO) described pseudo-grains as “the grains of the 21st century” because of their high nutritional value. In addition to their high complex carbohydrate content (around 63–82%), they are rich in protein (around 5–19%), have a favorable amino acid composition (sulfur-containing amino acids; methionine, cysteine, and lysine), and are an excellent source of dietary fiber (around 2.5–26.5%, mainly non-starchy polysaccharides, such as arabinoxylans and β-glucans) [12,86,93,94]. In addition to these, they also contain vitamins, minerals (e.g., calcium, iron, zinc), phytochemicals, polyphenolic compounds, betalains, and antinutrients (e.g., saponins, tannins, trypsin inhibitors, phytates, etc.) [94].

Because of their rich composition, pseudo-grains are attributed various properties, such as cholesterol-lowering, anti-inflammatory, blood pressure-lowering, anticancer, and liver-protective effects; thus, adequate consumption helps prevent and treat type 2 diabetes and obesity [10].

**Figure 3:**
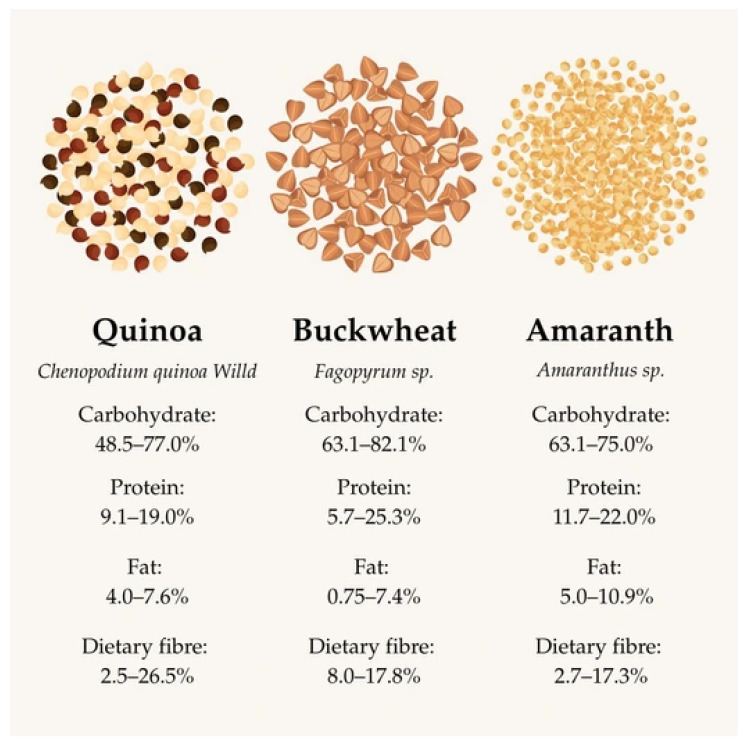
Nutrient content of pseudocereals according to their dry weight.

The nutritional values of the pseudo-grains are shown in Figure 3 [12,93,94].

#### 7.3.1. Carbohydrate Content

The carbohydrate content is about 50–80% of pseudocereals’ dry weight (quinoa: 48.5–77.0%, amaranth: 63.1–75.0%, buckwheat: 63.1–82.1%) [12,93,94].The highest proportion of starch content is found in quinoa (58.1–64.2%), followed by amaranth (65.0–75.0%) and buckwheat (54.5%). It is interesting to note that buckwheat has a higher starch content with a slower absorbing starch (amylose, 18.3–47%), than quinoa (11–12%) or amaranth (7.8–34.3%) in relation to the total starch content [12]. The variation in starch content depends on growth and climatic conditions [94]. In the case of pseudocereals, the resistant starch content should be highlighted. Unlike other starch types, resistant starches are not absorbed in the small intestine but are passed on to the large intestine where they are used as a nutrient by intestinal bacteria; thus, they not only keep blood sugar levels stable but also act as a prebiotic. This makes their consumption particularly beneficial in terms of the favorable diversity of the gut microbiome, thus reducing the incidence of colon cancer. According to the EFSA (European Food Safety Authority), starch-containing foods should contain a minimum amount of starch and should contain at least 14% resistant starch in relation to the total starch content [12,95]. Studies have shown that buckwheat species have the highest resistant starch content (27–33.5%) in pseudo-grains [12,96].

Simple carbohydrates are present in smaller proportions in pseudo-grains. Among the monosaccharides are glucose, fructose, arabinose, and xylose, while among the disaccharides, sucrose and maltose are typical. Quinoa and amaranth have a higher simple carbohydrate content (3–5%) than buckwheat (0.8%) and cereals (1–2%) [12]. The nutrient composition of different pseudocereals is shown in Table 5.

#### 7.3.2. Dietary Fiber Content

Pseudo-grains are an excellent source of dietary fiber. The majority of the fiber content is polysaccharides and lignin [10]. The total fiber content is 7.0–26.5% for quinoa, 2.7–17.3% for amaranth, and 17.8% for buckwheat. Furthermore, 78% of the total dietary fiber content in amaranth and quinoa is water-insoluble dietary fiber (e.g., homogalacturonate, rhamnogalacturonate-1, arabinan, xyloglucan, and cellulose), which primarily increase satiety, slow gastric emptying, and promote intestinal peristalsis through their effects on the intestinal wall. In addition, 22% of the total fiber content is water-soluble dietary fiber (e.g., another part of xyloglucan, pectin). Buckwheat contains a high proportion of total dietary fiber (17.8%), with a ratio of water-soluble to water-insoluble types ranging from 0.5 to 0.28. Among its fiber content, the cellulose, hemicellulose and lignin contents are noteworthy. The water-soluble fiber content is slightly lower for buckwheat, at 16.0% of the total fiber [12]. The fiber composition of buckwheat is composed of pectin (1.8%), hemicellulose (39.0%, including xyloglucan), lignin (20.0%), and cellulose (39.0%) [10]. The types of dietary fiber in pseudocereals are shown in Table 5.

#### 7.3.3. Protein Content and Amino Acid Profile

One of the main advantages of pseudocereals over cereals is their higher protein content [12]. According to some research, the protein values of pseudo-grains are comparable to one of the best proteins in terms of biological value, i.e., casein. In the case of pseudo-grains, the seed coat and the germ contain most of the proteins, with the rest being found in the perisperm or seed coat [94]. The protein content in this part of the grain is 9.1–16.7% for quinoa, 13.1–21.5% for amaranth and 5.7–14.2% for buckwheat [12]. The protein content is gluten-free and hypoallergenic. The protein composition includes globulins (salt-soluble proteins) and albumin (water-soluble proteins) [94]. The determining factor for protein content is the biological value, i.e., the presence of essential amino acids and the bioavailability of proteins. Pseudocereals have a higher methionine, lysine, and cysteine content than most cereals, thus ensuring amino acid balance [12]. The lysine content of quinoa is outstanding [12,99]. Amaranth is rich in lysine and tryptophan and has been shown to have health benefits when fortified with sorghum- and maize-based foods [94,100]. It is interesting to note that leucine was found to be the limiting amino acid in raw buckwheat flour, while lysine was the limiting amino acid in baked flour. In contrast, quinoa and amaranth have a high-quality amino acid profile that is particularly high in lysine, which is considered a limiting amino acid for cereals [11].

The amino acid profile of pseudocereals is shown in Table 5.

The quality of proteins is also a key factor in pseudo-grains, as quality metrics indicate the rate at which proteins are digested and utilized [12]. The biological value (BV) of pseudo-grains is higher than that of some cereals; quinoa has a BV of 73%, amaranth has a BV of 44.5–64.3%, and buckwheat has a BV of 90%. In terms of cereals, wheat has a BV of 55%, rice has a BV of 69,9%, and maize has a BV of 61% [10]. Interestingly, the biological value of buckwheat proteins was more than 90%, and because buckwheat is rich in essential and sulfur-containing amino acids, it achieved an amino acid score of 100 [10,100,101].

Using the PDCAAS method, the protein values of differently grown amaranths were determined to range from 23.7–36.2%, suggesting that amaranth can function as a suitable source of supplementary protein [12]. The PDCAAS values for quinoa, amaranth and buckwheat were 0.85, 0.70 and 0.78, respectively, in one study, while those for wheat, rice and maize were 0.42, 0.56 and 0.47, respectively [10].

According to DIAAS method, buckwheat (47.0–68.0 and 89.5) and quinoa (85.0) are better sources of protein for human consumption than oats, wheat 20.0), brown rice, polished rice (37.0), prosso millet, foxtail millet, maize (48.0) and barley pearl [10,12,93,102]. Table 6. shows the different values of protein digestibility methods (BV, PDCAAS, DIAAS) of pseudocereals according to previous research.

##### Quinoa

The protein content of raw quinoa varies between about 9.1–19.0% [12,87,93,94]. In general, storage proteins in cereals can be classified into four main groups, namely albumin (water soluble), globulins (soluble in dilute saline), prolamins (soluble in alcohol), and glutelins (soluble in alkaline media) [88], but with the highest proportion of prolamins. In contrast, quinoa contains less prolamin and glutelin (0.5–7% and 18%, respectively), but more globulin (37%) and albumin (35%). The main component storage protein is globular globulin, with molecular weights of 22–23 and 32–39 kDa. Quinoa is a good alternative for people with celiac disease because it has a low concentration of prolamin [88], which is an example of gliadin found in wheat [104]. As it does not contain gliadin, it is considered a gluten-free food and can be consumed by people with celiac disease [87].

Quinoa contains all the essential amino acids (phenylalanine, methionine, histidine, isoleucine, valine, leucine, lysine, threonine, and tryptophan) [87], most notably lysine (0.77–7.8 g/100 g protein), which is deficient in protein in most cereals. At the same time, it is also relatively high in threonine (0.42–8.9/100 g protein) and methionine (0.31–9.1/100 g protein) [10,12,87]. Quinoa has a protein efficiency similar to that of dairy casein [87].

##### Buckwheat

Buckwheat protein, like quinoa, is gluten-free due to the absence of α-gliadin and has a protein content of about 5.7–25.3% [12,93,94]. Most of the proteins are globulins, i.e., proteins that are soluble in salt [90]. The endosperm storage proteins are composed of 70% globulins, 25% albumin, 4% glutelins, and minimal prolamins (in comparison, glutelins and prolamins are more predominant in wheat) [85]. In terms of amino acid content, buckwheat contains a total of 12 amino acids, of which the lysine content (0.67–8.6 g/100 g protein) is noteworthy [10,12,87]. It also has a high content of sulfur-containing amino acids, e.g., methionine (0–17–2.5 g/100 g protein), cysteine (0.23–3.5 g/100 g protein), and is rich in arginine (0.98–11.3 g/100 g protein) [10,12,87,90].

##### Amaranth

The protein content is three times higher than that of maize [91]. It is around 11.7–22%, with albumin and globulins in the highest proportions [12,92,93,94]. The protein content of amaranth depends on food processing. Studies have shown that soaking and germination can boost the protein content by around 7%. Amaranth seeds have a higher protein content (16%) than cereals and pseudocereal seeds, but less protein than grains (24%) and chickpeas (19%) [92].

The amaranth grains are low in prolamin and do not contain α-gliadin, which makes amaranth flour suitable for celiac patients [91]. These fractions account for different percentages of the total protein, with albumins being the highest (11–52%), followed by globulins (16–51%), glutelins (7–36%), and prolamins (0–13%) [92].

The nutritional value of amaranth includes a high protein content and an essential amino acid composition that is superior to FAO/WHO standard requirements [37]. It has a balanced amino acid profile, contains all of the essential amino acids [37], and it is particularly high in lysine and methionine, but is also high in tryptophan [37,92]. The essential amino acids are leucine (0.88–6.9%), valine (0.68–5.0%), lysine (0.75–8.0%), threonine (0.56–5.0%), isoleucine (0.58-4.2%), phenylalanine (0.54–4.7%), methionine (0.23–4.6%), tryptophan (0.18–1.9%), and histidine (0.34–3.8%) [10,12,87,94].

Bioactive peptides can be produced during digestion in the gastrointestinal tract [94]. The protein fractions of different pseudocereals are shown in Table 7.

#### 7.3.4. Fat Content

According to studies, the fat content of quinoa is 4.0–7.6%, that of amaranth is 5.6–10.9%, and that of buckwheat is 0.75–7.4%. These results suggest that the lipid content of pseudo-grains is much higher than that of cereals. Their unsaturated fatty acid content is notable, ranging from 71.0–84.5% for quinoa, 61.0–87.3% for amaranth and 80.1–80.9% for buckwheat. This proves that the fat content of pseudocereals is variable [12].

Monounsaturated fatty acids are highest in buckwheat (35.7–47.9%), while omega-6 and omega-3 fatty acids are highest in quinoa (44.9–58.6% and 3.0–11.1%, respectively). Thus, the most abundant fatty acid in amaranth, quinoa, and buckwheat is linoleic acid, which accounts for about 50% of the total fatty acids in both amaranth and quinoa and about 35% in buckwheat [10,105]. The omega-6/omega-3 fatty acid ratio is also the highest for quinoa (4.7–19.6) [12].

It is peculiar that in quinoa and amaranth, polyunsaturated fatty acids (PUFAs), linoleic acid (ω-6 fatty acid), and alpha-linolenic acid (ω-3 fatty acid) account for 70–80% of the total fatty acid content, whereas in buckwheat, linoleic acid and oleic acid are the predominant fatty acids [12]. Quinoa contains 52% alpha-linolenic acid and 40% linoleic acid [12,94]. Table 5 shows the fat content according to saturation in pseudocereals.

#### 7.3.5. Mineral and Vitamin Content

Potassium, phosphorus, calcium, magnesium, zinc, and iron are also noteworthy in pseudocereals. The potassium content is the highest in quinoa (656–1475 mg/100 g), which makes it a prominent source of nutrients for cardiovascular diseases. Amaranth is the most abundant in calcium (175–206 mg/100 g). The highest values for magnesium were also found in quinoa (207.0–502.0 mg/100 g). In terms of iron content, values of 1.1–16.7 mg/100 g were measured for quinoa, 12.0–17.4 mg/100 g for amaranth, and 11.8–14.9 mg/100 g for buckwheat. The zinc content is the highest in amaranth (3.7–5.20 mg/100 g). Vitamins B1, B2, B3, B6, folic acid, vitamin E, and carotenoids (e.g., lutein, zeaxanthin, ß-carotene) are also present in quinoa. The vitamin B3 content of buckwheat (2.1–18.0 mg/100 g) and the vitamin E content of quinoa (24.7 mg/kg) are also worth mentioning [12].

#### 7.3.6. Biologically Active Compounds

Pseudocereals contain various phenolic compounds. In the case of quinoa, these include quercetin, kaempferol, ferulic acids, vanillic acid, rutin, daidzein, naringenin, apigenin, luteolin, myricetin, and catechin compounds [10]. The total phenolic content and total flavonoid content in quinoa ranged from 514.03 to 1409.54 mg gallic acid equivalent (GAE)/100 g and 177.49 to 407.75 mg rutin equivalent (RE)/100 g, respectively [103].

Amaranth contains the highest proportion of caffeic acid, rutin, ferulic acids, quercetin, and kaempferol. Buckwheat contains rutin, epicatechin, catechin, quercetin, and vitexin. These compounds have antibacterial, antifungal, antioxidant, anti-diabetic, anti-aging, neuroprotective, anti-inflammatory, and anti-proliferative activities in cancer [10].

## 8. Conclusions

The market for pseudocereal-based functional drinks is steadily growing. Research shows that functional foods and drinks are most popular because of their various health benefits. Functional beverages are available in a wide range of types and formulations, and can be categorized according to several criteria, groups, and classification systems. One such classification, presented in detail in this review, is based on the ingredients. The most common ingredient groups include water, vegetables, fruits, cereals, pseudo-grains, oilseeds, nuts, pulses, dairy products, milk replacers, plant-based beverages, eggs, tea, cocoa, coffee, fish oil, sea plants, spices, and herbs, as well as sweetening ingredients and biologically active substances (e.g., antioxidants, dietary fiber, prebiotics, probiotics, vitamins, minerals, etc.), each of which plays a very important role in promoting health. In recent decades, plant-based functional beverages have received a lot of attention as sources of plant protein and preferred choices in terms of sustainability, environmental concerns, ethical concerns, and the reduction of greenhouse gases.

In addition to being sustainable, plant protein sources are easy to digest, and many types are of very high quality and high in protein. Pseudo-grains, such as quinoa, buckwheat, and amaranth, are particularly good examples of this, as in addition to being high in fiber, slow-absorbing carbohydrates with unsaturated fatty acids and antioxidant activity, they are excellent sources of plant protein, which is almost their primary benefit. Research has shown that they contain all of the essential amino acids, are well utilized in the intestinal tract, have a high biological value, and are a high-quality source of protein compared to other cereals (e.g., rice, corn, and wheat). Therefore, pseudocereals can be a prime choice as main ingredients in plant-based functional beverages.

The number of studies on the exact amino acid composition is low, and research shows that there is a wide range of different amino acid values for different pseudocereals. This justifies the need for further specific research to precisely differentiate between the different quinoa, amaranth, and buckwheat varieties and to take into account the impact of different factors (e.g., climatic factors, type of cultivation, soaking or raw measurement, etc.). However, the results have shown that the essential amino acid content is indeed high for all pseudo-grains, with lysine and threonine being the most important ones.

## Figures and Tables

**Table 1:** Components of functional beverages.

Natural Sources	Type of Natural Sources	Bioactive Compounds	Nutritional Benefits of Natural Sources	References
Water	Natural water	-	Hydration, changes taste of functional beverages or the proportion of microbial species	[3]
	Mineral-enriched water	Calcium		
Vegetables	Carrot	ß-carotene, pectin, vitamin C, flavones	Anti-inflammatory and anticancer effects, antibacterial effects, improve digestion and promote regular bowel movements, support healthy gut microbiota, help to regulate blood sugar and cholesterol levels, boost immunity, support antioxidant status, vision, and skin health	[1,27,28,29,30,31,32]
	Tomato	Lycopene, trans-lycopene, pectin, anthocyanins,		
	Pepper	Capsaicin, pectin, flavones		
	Beetroot	Anthocyanins, iron, nitrates		
	Onion	Sulfur compounds, inulin		
	Garlic	Sulfur compounds, flavonols, lignans		
	Spinach	Vitamins A, C, and K, iron, calcium, zinc, magnesium, potassium, lutein		
	Curly kale	Dietary fibers, chlorophyll, flavonols, iron, calcium, zinc, magnesium, potassium		
	Chicory	Tannins		
	Rhubarb	Tannins		
	Pumpkin	ß-carotene, pectin,		
	Broccoli	Quercetin, selenium		
	Psyllium	Cellulose, hemicellulose, lignin		
Fruits	Red berries (raspberry, blueberry, blackcurrant, strawberry, blackberry, redcurrant)	Anthocyanins, dietary fiber (e.g., pectin), vitamin C, tannins	Favorable taste, support antioxidant status and immune function, anticancer and anti-inflammatory effects, protect against cardiovascular diseases and cognitive decline, improve digestion, support healthy gut microbiota	[1,3,9,15,27,33]
	Citruses (orange, lemon, lime, grapefruit)	Vitamin C, carotenoids, flavonoids		
	Red grapes	Resveratrol, quercetin, tannins		
	Kiwi	Prebiotic-compatible surfaces		
	Acai	Antioxidants		
	Cherry	Pectin		
	Peach	Pectin, tannins		
	Pomegranate	Anthocyanins, tannins		
	Pineapple	Bromelain, carotenoids, vitamin C		
Cereals	Oat	Manganese, phosphorus, magnesium, iron, ß-glucan	Improve antioxidant status, lowers LDL cholesterol levels, promote digestive health, help regulate blood sugar levels, vital for bone health, enzyme function, and energy production, anti-inflammatory and anticancer properties, versatility in food technology	[1,3,22,34]
	Wheat bran	Vitamin B1, dietary fiber (e.g., cellulose, hemicellulose, lignin)		
	Maize	Vitamin C, folic acid, cellulose		
	Malt	-		
Pseudocereals	Quinoa	Vitamins B1, B2, B3, C, and E, flavonoids (e.g., kaempferol, quercetin, ferulic acid, rutin)	Nutritional density, favorable protein composition, gluten-free, beneficial effects on digestive, metabolic, and cardiovascular functions, promote gut health and satiety, positive effect on the lipid profile (reduces levels of total cholesterol, triglycerides, and LDL cholesterol and increases levels of HDL cholesterol), reduce the incidence of type 2 diabetes, cancer, and cardiovascular disease, support antioxidant activity, help blood sugar control	[11,12,16,35,36,37,38]
	Buckwheat	Vitamins C, E, and B, rutin, quercetin, orientin, isoorientin and isovitexin		
	Amaranth	Vitamins C, E, and B, polyphenols, saponins, hemagglutinins, phytin, nitrates, oxalates, betacyanins (betanidine), rutin, isoquercetin, nicotiflorin		
Nuts and seeds	Flaxseed	Omega-3 fatty acid (alpha-linolenic-acid—ALA), vitamin E	Nutritional enrichment (protein), flavor and texture enhancers, play a role in lowering blood pressure, regulating cholesterol levels, supporting central nervous system health, improving memory, aiding digestion, promoting heart health, improving antioxidant activity, and helping blood sugar control	[1,22]
	Walnut	Omega-3 fatty acid (alpha-linolenic-acid—ALA), vitamin E		
	Almond	Monounsaturated fatty acids (e.g., omega-9 fatty acids), vitamin E		
	Cashew nut	Monounsaturated fatty acids (e.g., omega-9 fatty acids), vitamin E		
	Brazil nut	Selenium, monounsaturated fatty acids (e.g., omega-9 fatty acids), vitamin E		
	Sesame	Monounsaturated fatty acids (e.g., omega-9 fatty acids), vitamin E		
	Sunflower seeds	Omega-6 fatty acids, vitamin E		
Legumes	Soybeans	Isoflavones	Nutritional enrichment (protein), lower LDL cholesterol levels, improve cardiovascular health, support skeletal muscle health, increase satiety and support weight management, help regulate blood sugar levels, maintain bone density, reduce the risk of breast and prostate cancers, anti-inflammatory and anticancer properties	[1,22]
	Peas	Iron, highly bioavailabile protein, fiber, low allergenicity		
	Chickpeas			
Dairy products	Yoghurt	Probiotics, calcium, vitamin D	Improve digestion, boost immune function, and maintain the balance of the gut microbiome, improve bone density, enhance bone health, and reduce the risk of osteoporosis, complete protein source, support cardiovascular and cognitive health, anti-hypertensive properties	[1,3]
	Probiotic dairy products			
	Non-probiotic milk-based drinks (enriched with micronutrients)	Omega-3 fatty acids, vitamin D, magnesium, phosphorus		
	Whey protein isolate	Whey proteins		
	Fresh milk	Casein		
	Fermented milk	Probiotics		
Eggs	Egg yolk	Xanthophyll carotenoids (e.g., lutein, zeaxanthin)	Help muscle building, reduce the risk of cardiovascular disease, Alzheimer’s disease, and cancer, anti-hypertensive, antimicrobial, and antioxidant effects, contribute to immune protection, support eye, heart, and brain health	[1,39]
	Egg white	Ovalbumin		
	Egg protein hydrolysates	Hydrolyzed ovalbumin, antioxidants		
Fish and seafood	Omega-3 fatty acid-rich oil (from tuna, salmon or mackerel)	Omega-3 fatty acids (e.g., EPA, DHA), bio-calcium	Contribute to cardiovascular and joint health, reduce the risk of Alzheimer’s disease, support bone density, anti-hypertensive, anti-inflammatory, and antioxidant properties, promote beneficial microbiota and supports digestion (seaweeds)	[1,3]
	Seaweed	Prebiotics		
Herbs and spices and other plant sources	Turmeric	Diarylheptanoids, curcumin, dimethoxycurcumin, bisdimethoxycurcumin	Enhance natural flavor, anti-inflammatory, antimicrobial, anticancer, anti-diabetic, antioxidant, and neuroprotective effects, support digestibility and weight management, boost immunity, reduce the risk of heart disease, elevate mood, relieve stress	[1,3,9,40,41,42,43,44,45,46,47,48,49,50,51,52,53]
	Ginger	Zingiberene, beta-bisabolene, alpha-farnesene, beta-sesquiphellandrene, gingerol, paradol, shogaol		
	Fenugreek	Flavonoids, phenolic acids, coumarins, stilbenoids, tyrosol, pyrogallol, oleuropein, vanillic acid, ellagic acid, coumarin, quercetin, rutin, vitexin, isovitexin, and salicylates		
	Lemon balm	Ursolic acid, oleanolic acid, rosmarinic acid, quercetin, myricetin, epigallocatechin, and rutin, luteolin, caffeic acid		
	Peppermint	Terpene alcohol menthol, terpene ketone menthone, cineole, limonene, apigenin, luteolin, eriodictyol		
	Sea buckthorn	Gallic acid, vanillic acid, caffeic acid, ferulic acid, coumaric acid		
	Rosemary	Phenolic diterpenes, carnosic acid, carnosol, rosmarinic acid, rosmanol, epirosmanol, caffeic acid		
	Lavender	Linalool, linalyl acetate, cineol		
	Sage	Thujone, borneol, cineole, bornylesters, α-pinene, salvene, D-camphor phellandrene, ocimene, rosmarinic acid, carnosic acid, carnosol, rosmanol		
	Mahua	Madhucic acid, erythrodiol, oleanolic acid, betulinic acid, quercetin		
	Thyme	Thymol, carvacrol, linalool, L-borneol, geraniol, amyl alcohol, β-pinene, camphene, p-cymene, caryophyllene, 1,8-cineole, rosmarinic acid		
	Rosehip	Vitamins C and E, vanillic acid, coumaric acid, vanillin, sinapic acid		
	Fennel	Trans-anethole, fenchone, and estragole		
	Lemon grass	Limonene, linalool, citronellal, isoneral, citronellol, neral, geraniol, geranyl acetate, geranial		
	Green tea	Catechins (e.g., epigallocatechin gallate—EGCG)		
	Cocoa	Carotenoids, catechins, anthocyanin, theobromine		
	Coffee, coffee leaves	Theophylline, quercitrin, isoquercitrin, kaempferol, chlorogenic acids, mangiferin, iso-mangiferin, rutin, tannins, caffeic acid, caffeine, trigonelline, related glycosides		
Sweetening ingredients	Naturally sweetened	-	Provides a sweet taste, natural source of sugar, moderates blood sugar levels, supports digestion and gut health, anti-inflammatory effects, synergistic actions in beverages (improves their sensorial properties and stability)	[3,54,55,56,57,58]
	-	Mannitol		
	-	Lactulose		
	Dates, date syrup	Natural sugars, cellulose, pectin, antioxidants, phenolic compounds		
	Honey	Polyphenols (flavonoids)		
	Agave syrup	Fructose, sucrose, kestose, inositol, fructo-oligosaccharides, inulin		

**Table 3:** Vitamins and vitamin sources.

Vitamins	Vitamin Sources
Vitamin A	Green leafy, orange, and yellow vegetables, such as carrots and spinach
Vitamin B1	Whole grains, meat, and fish
Vitamin B2	Eggs, organ meats, lean meats, and milk
Vitamin B3	Meat, poultry, fish, fortified and whole grains, mushrooms, potatoes
Vitamin B12	From animals but not plants (e.g., dairy product, eggs)
Folic acid	Grain-based products
Vitamin C	Citrus fruits (e.g., oranges, grapefruit) and their juices, red and green pepper, kiwifruit, broccoli, strawberries, baked potatoes, tomatoes
Vitamin D	Fish oil-fortified foods (e.g., milk)
Vitamin E	Vegetable oils (such as wheat germ, sunflower, and safflower oils), nuts (such as almonds), seeds (such as sunflower seeds), and green vegetables (such as spinach and broccoli)
Vitamin K	Green leafy vegetables, berries
**Minerals**	**Mineral Sources**
Calcium	Milk, cheese, and yogurt; vegetables, like kale, broccoli, and Chinese cabbage; canned sardines and salmon with soft bones, nuts and seeds, legumes, tap water, mineral water
Iron	Lean meat, seafood, poultry, beans
Magnesium	Legumes, nuts and seeds, whole-wheat bread, dairy products
Phosphorus	Dairy products, milk
Zinc	Red meat, poultry, oysters and other seafood, cereals, beans, nuts, whole grains, and dairy products

**Table 4:** Physiological functions of dietary fibers.

Physiological Effect	Disease	Reference
Provides the mass of the feces and improves intestinal motility	Constipation and other gastrointestinal disorders, colorectal cancer	[79]
Delays the emptying of the stomach	Obesity	
Improves the intestinal absorption of glucose	Type 2 diabetes	
Increases the excretion of fats	Cardiovascular diseases	
Reduces the degree of dysbiosis	Cardiometabolic diseases, colorectal cancer, mental health, immune health	
Ensures the continued integrity of the intestinal lining	Metabolic alterations, immune health	
Regulates enteroendocrine function	Obesity, type 2 diabetes	
Improves gene expression	Cardiovascular diseases	
Modulates amino acid metabolic signatures	Type 2 diabetes	

**Table 5:** Nutrient composition of different pseudocereals.

Nutrients	Quinoa	Buckwheat	Amaranth	References
***Carbohydrate*** **(% of dry basis)**	48.5–77.0%	63.1–82.1%	63.1–75.0%	[12,93,97]
Starch	52.2–69.2%	54.5–57.4%	65.0–75.0%	[10,12,97]
Amylose (% of total starch)	11.0–12.0%	18.3–47%	7.8–34.3%	
Resistant starch (% of total starch)	-	27.0–33.5%	-	
Simple carbohydrate (mono- and disaccharides)	3.0–5.0%	0.8%	3.0–5.0%	
***Dietary fiber*** **(% of dry basis)**	2.5–26.5%	8.0–17.8%	2.7–17.3%	[10,12,93,94,97]
Water-soluble dietary fiber	22.0%	16.0%	14.0–22.0%	[10,12,97]
Pectin	-	1.8%	-	
Xyloglucan	30.0%	-	-	
Water-insoluble dietary fiber	78.0%	70.3%	78.0–86.0%	
Hemicellulose	-	39.0%	-	
Lignin	-	20.0%	-	
***Protein*** **(% of dry basis)**	9.1–19.0%	5.7–25.3%	11.7–22.0%	[12,93,94,97]
*Essential amino acids* *(g/100 g of total protein)*				
Histidine	0.41–5.4	0.29–4.9	0.34–3.8	[10,12,81,97]
Isoleucine	0.50–7.4	0.49–4.1	0.58–4.2	[10,12,81,97]
Leucine	0.84–9.4	0.83–7.6	0.88–6.9	[10,12,81,97]
Lysine	0.77–7.8	0.67–8.6	0.75–8.0	[10,12,81,97]
Methionine	0.31–9.1	0.17–2.5	0.23–4.6	[10,12,81,97]
Phenylalanine	0.59–4.7	0.52–7.2	0.54–4.7	[10,12,81,97]
Threonine	0.42–8.9	0.51–4.0	0.56–5.0	[10,12,81,97]
Tryptophan	0.17–1.9	0.19–1.83	0.18–1.8	[10,12,81,97]
Valine	0.59–6.1	0.68–6.1	0.68–5.0	[10,12,81,97]
*Non-essential amino acids* *(g/100 g of total protein)*				
Alanine	0.58–5.7	0.75–9.6	0.78–6.2	[10,12,81,97]
Arginine	0.03–13.6	0.98–11.3	1.06–15.6	[10,12,81,97]
Asparagine	0.35	-	-	[98]
Aspartic acid	1.13–8.0	1.13–16.6	1.26–10.0	[10,12,81,97]
Cysteine	0.19–2.7	0.23–3.5	0.19–3.6	[10,12,81,97]
Glutamine	0.68	-	-	[98]
Glutamic acid	1.86–13.2	2.05–24.4	2.26–17.7	[10,12,81,97]
Glycine	0.30–6.1	1.03–13.2	1.64–15.2	[10,12,81,97]
Proline	0.77–5.5	0.5–8.8	0.69–4.6	[10,12,81,97]
Serine	0.47–5.7	0.68–8.6	1.15–9.3	[10,12,81,97]
Tyrosine	0.27–3.7	0.24–4.9	0.33–3.7	[10,12,81,97]
***Fat*** ***(% of dry basis)***	4.0–7.6%	0.75–7.4%	5.0–10.9%	[10,12]
Unsaturated fatty acids (% of total total lipid)	80.1–80.9%	70.0–89.4%	61.0–87.3%	[10,12]
*Monounsaturated fatty acids* *(g/100 g of total fat)*	1.610	1.040	1.680	[10]
*Palmitoleic acid (C16:1)* *(% of total lipid)*	-	0.15–0.20%	-	[12]
Oleic acid (C18:1–9c) (% of total lipid)	15.7–31.1%	35.7–47.9%	18.7–38.9%	[10,12]
Vaccenic acid (C18:1–11c) (% of total lipid)	1.3–1.7%	-	1.4–2.00%	[12]
Gondoic acid (C20:1) (% of total lipid)	0.6–1.6%	1.8–3.1%	0.2–0.3%	[12]
Erucic acid (C22:1) (% of total lipid)	1.5%	0.2–0.5%	0.1%	[12]
*Polyunsaturated fatty acids* *(g/100 g of total fat)*	3.290	1.040	2.780	[10]
Linoleic acid (C18:2) (% of total lipid)	38.9–58.6%	31.4–44.6%	33.0–55.9%	[10,12]
α-Linolenic acid (C18:3) (% of total lipid)	3.0–11.1%	0.0–5.3%	0.2–1.97%	[10,12]
*Saturated fatty acids (% of total lipid)*	15.5–29.0%	18.8–19.5%	20.1–30.9%	[10,12]
Lauric acid (C12:0)	-	0.02–0.04%	-	[12]
Myristic acid (C14:0)	-	0.07–0.1%	-	[12]
Pentadecylic acid (C15:0)	-	0.05–0.06%	-	[12]
Palmitic acid (C16:0)	9.3–10.7%	13.2–18.5%	18.8–20.2%	[12]
Margaric acid (C17:0)	-	0.05–0.06%	-	[12]
Stearic acid (C18:0)	0.7–1.1%	1.4–6.3%	3.7–4.2%	[12]
Arachidic acid (C20:0)	-	1.1–1.2%	-	[12]
Behenic acid (C22:0)	-	1.1–1.3%	-	[12]
Lignoceric acid (C24:0)	-	0.7–0.8%	-	[12]

**Table 6:** Protein digestibility values of pseudocereals.

Method	Buckwheat	Amaranth	Quinoa	References
BV (%)	90%	44.5–64.3%	73%	[10]
PDCAAS (score)	0.78	0.70	0.85	[10]
DIAAS (score)	47.0–89.5	-	85.0	[10,12,83,102]
In vitro protein digestibility (%)	-	79.19%	80.49%	[83,103]

**Table 7:** Protein fractions of different pseudocereals.

Types of Proteins	Buckwheat	Amaranth	Quinoa	References
Albumins	25%	11–52%	35%	[85,88,92]
Globulins	70%	16–51%	37%	
Prolamins	Minimal	0–13%	0.5–7%	
Glutelins	4%	7–36%	18%	

## Data Availability

The original contributions presented in the study are included in the article. Further inquiries can be directed to the corresponding author.
